# Risk Factors for Cognitive Impairments in Chronic Cannabis Use: A Systematic Review

**DOI:** 10.1192/j.eurpsy.2026.10497

**Published:** 2026-07-31

**Authors:** H. Hsine, W. Abbes, A. Bouaziz, S. Kolsi, M. El Behi

**Affiliations:** 1 Psychiatry, La Metairie Clinic, Nyon, Switzerland; 2 Psychiatry, Gabes university Hospital, Gabes, Tunisia

## Abstract

**Introduction:**

Chronic cannabis use has been associated with various factors that may contribute to the development of sensory and cognitive impairments.

**Objectives:**

This review aims to explore the main risk factors linked to the emergence of such deficits in the context of long-term cannabis consumption.

**Methods:**

A systematic review of the literature was conducted, analyzing nine articles following the PRISMA-P methodology. The literature search was performed across the Cochrane Library, PubMed, Embase, and PsycINFO databases, covering the period from 2014 to 2024. The included studies synthesized available data on risk factors related to the onset of cognitive impairments following chronic cannabis use.

**Results:**

Among the 30 studies identified, 9 fulfilled the inclusion criteria. Cognitive alterations appear to be influenced by multiple factors, including early initiation (before the age of 16), high frequency and long duration of use, high THC potency, genetic vulnerability (family history of schizophrenia), environmental stressors (such as stress, poverty, and low educational level), and gender (with some evidence suggesting greater sensitivity among females). Concurrent use of other substances may also exacerbate cognitive effects.

**Conclusions:**

The severity and persistence of cannabis-related cognitive impairments are attributable to a range of interrelated factors. Early identification of these risk factors could help prevent long-term cognitive harm associated with regular and prolonged cannabis use.

**Disclosure of Interest:**

None Declared

